# Survey of motivation for use of voluntary counseling and testing services for HIV in a high risk area of Shenyang, China

**DOI:** 10.1186/1472-6963-9-23

**Published:** 2009-02-05

**Authors:** Ling Zhou, Junqiao Guo, Lijuan Fan, Jing Tian, Baosen Zhou

**Affiliations:** 1 Department of Epidemiology, School of Public Health, China Medical University, Shenyang, PR China; 2 Liaoning Provincial Center for Disease Control and Prevention, Shenyang, PR China; 3 Heping County Center for Disease Control and Prevention, Shenyang, PR China; 4 Heping County board of health, Shenyang, PR China

## Abstract

**Background:**

HIV voluntary counseling and testing (VCT) is considered an effective prevention method of HIV infection. In order to understand the VCT environment and enhance the effective delivery of VCT services in a country, an accurate assessment of the current status of VCT services is very important.

**Methods:**

From July 2006 to June 2007, we conducted a cross-sectional survey using a face to face interview among 2676 VCT clients from a high risk area in Shenyang city, China.

**Results:**

The major demographic characteristics among 2,676 VCT clients were: 41.1% were in the age range 20 to 30 years; 73.1% were males; and 67.1% had attained the level of junior high school education. The primary information source for VCT services was mass media like television (TV) and newspaper in 88.9%. 34.3% were afraid of the result of infection which was the main barrier to accept VCT services among 540 participants answering the question. 75.2% were motivated by recently acquired knowledge about HIV. 47.9% had 3 or more male sex partners, 62.3% had used condoms sometimes, and 14.5% had been infected with a STD. 2.8% of the participants identified themselves as men who have sex with men (MSM). The main demographic characteristics of MSM did not differ from the total group of participants except with respect to age: 63.5% reported having one male sex partner in the preceding 12 months, 44.6% reported never using condoms in the preceding 12 months, and only 2.7% reported a history of sexually transmitted disease.

**Conclusion:**

Public education offered by health workers in hospitals, private clinics and other medical institutions needs to be strengthened. Given the results from this study, we recommend: (1) making VCT a routine part of health services, especially in areas where many high-risk individuals live; (2) improving the information sources and increasing the understanding of HIV and HIV-infected individuals; (3) enhancing international collaboration in strategic planning, technical assistance, and protocols to translate policy into effective action; (4) supporting Chinese non-government organizations (NGOs) in playing a significant role in the battle against AIDS.

## Background

There are some 37.8 million people living with HIV (human immunodeficiency virus)/AIDS (acquired immunodeficiency syndrome) globally of which about 7.4 million live in Asia. Around one million HIV positive people live in China, according to official estimates [1]. Currently, China has the second highest number of people living with HIV/AIDS (PLHA) in Asia and is ranked 14th in the world [2]. The national estimate of the number of PLHA at the end of 2003 was 840,000 (650,000–1,020,000), with a prevalence rate in the general population of 0.07% (0.05%–0.08%). The UN estimates that China may have 10 million people infected with the HIV virus by 2010 unless effective action is taken in 2002 [3].

Shenyang city is the capital of Liaoning Province, the largest and most industrialized city in Northeast China. Xita area is located in the center of Shenyang and has communicated largely with other countries in economy and culture. Xita area occupies 0.58 square kilometers, with a total population of 23,167, of which 5893 are of the Korean ethnicity. It has a floating population of 3,600 who is temporary rural-urban labor migration. There are 207 entertainment places, such as pubs, hairdressing salons, bathing centers, massage parlors, song bars, hotel and ballrooms. Xita area has the world's second largest ex-patriate Korean community, after Los Angeles, USA [4]. With economic development, Xita area has become an area with high incidence of HIV/AIDS. In 2003, the Chinese government established 51 HIV comprehensive prevention and care model points countrywide. The only point in Shenyang is situated in Xita, because it harbours a substantial number of high risk populations like injecting drug users, commercial sex workers and MSM who have been identified as high populations of HIV/AIDS in China [5-7].

The number of MSM in China was estimated at between 2 to 8 million [6,8], and 47,000 MSM were estimated to be living with HIV/AIDS, accounting for 7.3% of the total number of estimated HIV cases by the end of 2004 in China [9]. Most of the known MSM population resides in the metropolitan areas. MSM can be divided into three categories: homosexual, heterosexual and bisexual. Unprotected sexual activities, including group sex, anal sex, casual sex and commercial sex are prevalent among Chinese MSM [10]. A study published in 2006 revealed that 41% Of MSM had engaged in sex with women as well as men, including married women [10]. MSM are at high risk of HIV infection and contribute to the spread of HIV to women and the general population. In addition, groups of male commercial sex workers who have sex with men only for money have appeared in many large cities.

A policy was developed to combat HIV/AIDS in China known as the "Four-free plus care": (1) free antiretroviral (ARV) drugs to AIDS patients who are rural residents, or people with financial difficulties living in urban areas; (2) free VCT in areas where the disease is prevalent; (3) free ARV drugs to HIV infected pregnant women to prevent MTCT, and HIV testing of newborns babies; (4) free schooling for children orphaned by AIDS; and (5) care and economic assistance to the households of people living with HIV/AIDS. VCT has been described as an important intervention for HIV prevention as it may serve as an entry point for prevention and care and support for those found infected [11,12]. It is considered an effective method of prevention of HIV infection, as well as improves the quality of care and assistance to HIV infections [13]. By the end of 2005, there were 2,850 free VCT clinics throughout the country [9].

In order to enhance the effective delivery of VCT services in a country, an accurate assessment of the current status is very important. A study by Weiser et al. in Botswana among people who were never tested for HIV, showed that the major reason for not testing was fear of a positive HIV test result [14]. Jereni et al. surveyed clients of VCT in Malawi to assess their motivations for HIV testing. They found that obtaining knowledge about HIV recently was the main motivation for seeking VCT [13]. The same result was found by Sherr et al. [15]. Perceived risk of HIV infection had a major influence on VCT readiness among young people in an urban population in Zambia [16]. Low perceived risk for HIV infection is major reason for ignoring HIV testing and counseling in some areas [17,18].

Information sources, barriers and motivations for seeking VCT services vary from place to place and could vary from time to time. The aims of this study were: (1) to understand the demographic and behavioral characteristics of the individuals seeking VCT services in Xita area; (2) to describe the demographic and behavioral characteristics of MSM among those seeking VCT services; (3) to find effective means of communication and service, and improve the effectiveness of VCT system, through the study of information sources, barriers and motivations for seeking VCT service.

## Methods

This was a cross sectional study utilizing a standardized interview questionnaire designed for the survey to assess information sources, barriers, and motivations of VCT.

### Participants

We selected Xita area which is one of 51 community-based HIV/AIDS comprehensive care pilot centers in regions with the greatest number of HIV/AIDS cases. The participants were the VCT clients from July 2006 to June 2007. The Chinese government owned the Korean National Hospital and Maternity and Child Care Centers provided VCT services. Providing VCT service is only a partial function of the two facilities. The minimum age to take VCT without parental consent is 18 years.

Inclusion criteria in the study were: (1) the age range 20 to 65 years; (2) willingness to participate in the interview after detailed explanation by interviewers. Informed consent was obtained before the interview. MSM were defined as men had sex with men in the past one year. We invited 7517 VCT clients who were in the age range 20 to 65 years to participate in the study, 35.6% agreed to been interviewed.

### Data collection

The study instrument included a 15-min questionnaire, which was designed for the survey and administered face-to-face to all participants. The interview points were the department providing VCT services of the Korean National Hospital and Maternity and Child Care Centers. The interview occurred before testing. The questionnaire included demographic and behavioral characteristics, information sources, barriers, and motivations of VCT. MSM and other participants were interviewed using the same questionnaire. The health workers who provided VCT services were trained to take the interview. The questions about barriers explored why the participants had never been tested before they presented at the VCT clinic.

### Data analysis

Quantitative data were entered into and analyzed using Epi Info software. We also performed chi-square test to assess any differences in responses between males and females, MSM and the general population. A p value of < 0.05 was considered statistically significant.

### Ethical issues

This study was approved by the Ethics Committee of China Medical University. The study was done under the full knowledge and consent by participants, who were assured of the security of the information. Written, informed consent was received from all study participants before the HIV testing. All study participants received a soft drink during the interview and gifts after the interview. The gifts were HIV prevention handbooks, nail-clippers and a 40 Yuan coupon for free testing and counseling services at designated facilities.

## Results

### The demographic characteristics of participants

Socio-demographic characteristics are presented in Table 1. There were 2,676 participants in the survey. Of the participants seeking VCT during the study period in Xita, 41.1% were in the age range of 20 to 30 years, and the majority (67.1%) had attained the level of junior high school. 73.1% were males while 26.9% were females. Most were married (59.3%) followed by single participants (26.2%) and divorced participants (14.5%). The majority was of the Han ethnicity (82.9%).

**Table 1:** Demographic characteristics of the participants

Characteristic		Total		MSM ^a^	
		Number	%	Number	%
Age	20–30	1099	41.1%	21	28.4%
	31–40	1067	39.9%	36	48.6%
	41–50	403	15.1%	12	16.2%
	51–65	107	4.0%	5	6.8%
Sex	Male	1957	73.1%	74	100%
	Female	719	26.9%	-	-
Ethnicity	Han	2219	82.9%	64	86.5%
	Korean	432	16.1%	7	9.5%
	Other	25	0.9%	3	4.1%
Education Level	Elementary school/none	53	2.0%	2	2.7%
	Junior high school	1795	67.1	45	60.8%
	Senior high school	602	22.5%	19	25.7%
	College	226	8.4%	8	10.8%
Marital Status	Single	701	26.2%	12	16.2%
	Married	1586	59.3%	51	68.9%
	Separated/divorced	389	14.5%	11	14.9%
Sexual self-identity	Homosexual	0	0	0	0
	Heterosexual	2674	99.93%	72	97.3%
	Bisexual	2	0.07%	2	2.7%

Total		2676	100%	74	100%

a MSM were extracted from 'total' and 'males'

### Information sources of participants

Information sources, stratified by gender and sexual orientation are presented in Table 2. There were 2,623 (98.0%) participants answering this question in Xita. 88.9% acquired the VCT services information from media reported as the most common source, such as TV, newspaper and radio. Females were more likely to acquire information from the public education by community or Red Cross and the recommendation offered by health workers than males, p < 0.05.

**Table 2:** Information sources of the participants

Source	Total (%)	Males (%)	Females (%)	MSM ^a ^(%)
Media-TV, newspaper and radio	2380(88.9)	1733 (88.6)	647(90.0)	64(86.5)
Public education by community or Red Cross	1134(42.4)	829(42.4)	305(42.4)*	29(39.2)
Recommendation offered by health workers	315(11.8)	230(11.8)	85 (11.8)*	13(17.6)
Friends/family	722(27.0)	533(27.2)	189(26.3)	19(25.7)
Internet	1030(38.5)	744(38.0)	286(39.8)	33(44.6)
Others	494(18.5)	378(19.3)	116(16.1)	15(20.3)

a MSM were extracted from 'total' and 'males'

* Significantly different between males and females (p < 0.05)

### Barriers to VCT

Participants indicated whether any of the listed factors served as a barrier for them (Table 3). They could agree with multiple possible responses. Of the 540 participants answering the question in Xita, 185 participants were afraid of the result of infection which was the main barrier to accept VCT service. There was no difference in barriers to VCT between males and females.

**Table 3:** Barriers to VCT

Barrier	Total (%)	Males (%)	Females (%)	MSM ^a ^(%)
Fear of positive test result	185(6.9)	147(7.5)	38(5.3)	4(5.4)
Stigma/Discrimination on HIV/AIDS	34(1.3)	19(1.0)	15(2.1)	1(1.4)
Fear of losing job, family or friends	82(3.1)	62(3.2)	20(2.8)	4(5.4)
Fear of hospital	56(2.1)	43(2.2)	13(1.8)	2(2.7)
Worried someone would find out about test result	1(0)	0(0)	1(0.1)	0(0)
Didn't have time	56(2.1)	39(2.0)	17(2.4)	0(0)
Others	169(6.3)	122(6.2)	47(6.5)	8(10.8)

a MSM were extracted from 'total' and 'males'

### Motivations for seeking VCT services

Two thousand and eighty-five (77.9%) participants answered this question (Table 4). Of these, 2,013 participants (75.2%) considered that recent knowledge about HIV was the main motivation. Males and females did not differ in the various motivations except recent knowledge about HIV.

**Table 4:** Motivations for seeking VCT services

Motivation	Total (%)	Males (%)	Females (%)	MSM ^a ^(%)
Feeling the symptoms of HIV	112(4.2)	91(4.6)	21(2.9)	3(4.1)
Perception of own high risk behavior	31(1.2)	25(1.3)	6(0.8)	0(0)
Recently acquired knowledge about HIV	2013(75.2)	1473(75.3)	540(75.1)*	54(73.0)
Confirmatory test	1(0)	1(0.1)	0(0)	0(0)
Other	157(5.9)	117(6.0)	40(5.6)	5(6.8)

a MSM were extracted from 'total' and 'males'

* Significantly different between males and females (p < 0.05)

### Behavioral characteristics of participants

47.9% had 3 or more sex partners, 62.3% participants had used condoms sometimes, and 14.5% had been infected with a STD.

### The demographic characteristics, behavioral characteristics, information sources, barriers and motivations for MSM who sought VCT services

Seventy-four persons were MSM in the survey. The main demographic characteristics of MSM did not differ from the entire participants, except for age (Table 1). The majority (48.6%) were in the age range 31 to 40 years. Forty-seven (63.5%) reported having one male sex partner in the preceding 12 months (Table 5). A total of 33 (44.6%) participants reported never using condoms in the preceding 12 months and only two (2.7%) reported a history of a sexually transmitted disease (STD).

**Table 5:** The behavioral characteristics of the participants

Characteristic		Total (%)	MSM ^a ^(%)
Past year condom use	Sometimes	1667(62.3%)	20(27.0%)
	Always	354(13.2%)	21(28.4%)
	None	655(24.5%)	33(44.6%)
Number of sex partners in past year	1	256(9.6%)	47(63.5%)
	2	1136(42.5%)	16(21.6%)
	≥ 3	1284(47.9%)	11(14.9%)
History of sexually transmitted disease	Yes	389(14.5%)	2(2.7%)
	No	2287(85.5%)	72(97.3%)

Total		2676(100%)	74(100%)

a MSM were extracted from 'total' and 'males'

There were no difference between MSM and the whole participants in the major information sources and motivations. The most commonly identified barrier for the whole sample was fear of the test result, whereas the most common barrier for MSM was "other".

## Discussion

To our knowledge, this is the first study about information, barriers and motivations regarding free VCT in an area with a relatively high prevalence of HIV in China. These findings are useful in the development of more effective VCT services and intervention and prevention programmes in China.

We found that 41.1% of participants were in the age range 20 to 30 years and 39.9% were in the age range 31 to 40 years. We believe this could be explained by a perception that young adults may feel more at risk due to their own sexual behaviors compared to older adults. It is also possible that the young have more comprehensive information sources than older adults because they have a larger scope of activities. Conversely, it is also plausible to consider that younger individuals have a higher degree of acceptance than older ones toward unfamiliar things. Jereni et al. reported that the majority of clients seeking VCT services were in the age range 20 to 30 years, men, the level of junior high school and married [13]. Ma W et al. reported that half of the participants were in the age range 30 to 40 years [18]. In the study by Fylkesnes et al., readiness was higher in younger than older age groups, e.g. 49% in the age range 20 to 24 years versus 23% in the age range 40 to 49 years [16]. This is in concordance with our findings.

Among information sources for VCT, the most popular one was mass media such as TV, newspaper and radio. This is most likely the result of the activities of the State AIDS Working Committee that was created in 2004 to ensure effective implementation of the State Council Document Number 7. The leaders of this committee have promoted and implemented education programs. The campaigns included: creating television shows that teach people about AIDS/HIV; inviting celebrities to make public service announcements about AIDS/HIV on TV shows or in newspaper; creating teaching materials for secondary school students and posting advertisements in public spaces, such as train and subway stations [7]. The area covered by TV or newspapers is far larger than any of the other communication media. In Guizhou province, Ma W et al. reported that the decision to be tested for HIV was influenced by the people around them [18]. In Nigeria, mass media and churches were the most common sources of information on VCT [19]. We found that information obtained from professional sources was minimal, indicating an opportunity to improve health education offered by health workers in hospitals, private clinics and other medical institutions.

Among barriers to VCT, the perception of fear of a positive HIV test result was the most common reason for not being tested. In addition, no-one indicated that they felt "worried other people would be told test results without consent" was a barrier to them. The fear of discrimination represents an important barrier to testing and counseling [20,21]. A potential explanation for this inconsistency with previous studies is that different geographical regions, cultures and economic levels have different attitudes toward VCT. Another possibility is that the subjects of this investigation were voluntary, while the subjects in the literature were individuals who didn't seek VCT services.

Our study also showed that recent knowledge about HIV was the major motivation for VCT. This result was similar to a study by Jereni et al. in Malawi [13]. In a study from Zimbabwe, Morin et al. reported that individuals who perceived themselves as being at high risk of HIV infection were more likely to present at mobile HIV testing sites [22]. That was the major motivation for seeking VCT.

No one of the MSM identified himself as being homosexual. A study from the USA reported that 79.7% identified as homosexual [23]. The reason of the significant differences with our study may be that MSM in China may be ashamed and stigma of their sexual identity and therefore conceal it deliberately.

The study has certain limitations. Firstly, the questionnaire contained some sensitive questions which could have led to low response rate or information bias. Secondly, we recruited only a small number of MSM, which may not be representative of Chinese MSM. There is need for a future studies with larger sample sizes among MSM in order to validate our findings. In addition, as the sample included only those voluntarily accessing services, the results can also not be extrapolated to the general population.

## Conclusion

Public education offered by health workers in hospitals, private clinics and other medical institutions needs to be strengthened. The VCT system also needs to pay attention to education about HIV and VCT. Given the results from this study, we recommend: (1) making VCT a routine part of health services, especially in areas where many high-risk individuals reside; (2) improving the information sources and increasing the understanding of HIV and HIV-infected individuals; (3) enhancing international collaboration in strategic planning, technical assistance, and protocols to make policy into effective action; (4) supporting Chinese non-government organizations (NGOs) in playing a significant role in the battle against AIDS.

## Competing interests

The authors declare that they have no competing interests.

## Authors' contributions

LZ performed the statistical analysis, drafted and revised the manuscript. JG conceived of the study, and participated in its design and data collection. BZ carried out the design, data analysis and interpretation. LF and JT participated in data collection. All authors read and approved the final manuscript.

## Pre-publication history

The pre-publication history for this paper can be accessed here:


